# Management of the Clinically N0 Neck in Maxillary Squamous Cell Carcinoma: A Systematic Review and Meta-Analysis

**DOI:** 10.3390/jcm15114310

**Published:** 2026-06-02

**Authors:** Fabio Maglitto, Giovanni Salzano, Serena Trotta, Eutilia Manzo, Luigi Angelo Vaira, Umberto Committeri, Stefania Troise, Giovanni Dell’Aversana Orabona

**Affiliations:** 1 Maxillofacial Surgery Unit, Department of Neurosciences, Reproductive and Odontostomatological Sciences, University of Naples Federico II, 80131 Naples, Italy; fabio.maglitto@aoufedericoii.unina.it (F.M.); giovannisalzanomd@gmail.com (G.S.); eutilia.manzo@libero.it (E.M.); stefania.troise@unina.it (S.T.); giovanni.dellaversanaorabona@unina.it (G.D.O.); 2 Maxillofacial Surgery Unit, University Hospital of Sassari, 07100 Sassari, Italy; lavaira@uniss.it; 3 Department of Maxillofacial Surgery, “S. Maria” Hospital, 05100 Terni, Italy; umbertocommitteri@gmail.com

**Keywords:** maxillary neoplasms, squamous cell carcinoma, neck dissection, elective neck dissection, occult metastasis, survival, meta-analysis

## Abstract

**Background:** The optimal management of the clinically node-negative (cN0) neck in maxillary squamous cell carcinoma (MSCC) remains controversial due to the relatively low yet clinically relevant risk of occult cervical metastasis. While elective neck dissection (END) has been proposed to improve oncologic outcomes, others advocate observation to avoid overtreatment. This study aimed to evaluate the impact of END versus observation on overall survival (OS) in cN0 MSCC patients. **Methods:** A systematic literature search was conducted in PubMed, Embase, and Scopus according to the PRISMA 2020 statement. The protocol was registered in PROSPERO (CRD420261345038). Studies including patients with maxillary SCC and clinically N0 neck comparing END with observation were eligible. Fourteen studies were included in the qualitative synthesis, and five were suitable for quantitative meta-analysis. The primary endpoint was OS. Pooled hazard ratios (HRs) with 95% confidence intervals (CIs) were calculated using a random-effects model. Risk of bias was assessed using ROBINS-I, and certainty of evidence was evaluated using the GRADE framework. **Results:** The meta-analysis demonstrated a statistically significant survival benefit associated with END. The pooled HR for OS was 0.76 (95% CI 0.67–0.86; *p* < 0.001), indicating a 24% relative reduction in the hazard of death compared with observation. Importantly, statistical heterogeneity was negligible (I^2^ = 0%), although interpretation should be cautious given the limited number of included studies. Despite this, most included studies were retrospective in design. **Conclusions:** END appears to provide a survival advantage in selected patients with cN0 MSCC. However, the evidence is largely derived from retrospective data. High-quality prospective multicenter studies are needed to better define the role of elective neck management in this population.

## 1. Introduction

Squamous cell carcinoma (SCC) arising from the maxillary subsites, including the maxillary gingiva, alveolus, hard palate, and maxillary sinus, represents a relatively uncommon subset of head and neck malignancies. Despite its lower incidence compared with other oral cavity tumors, maxillary SCC often presents at an advanced stage and poses unique challenges in regional disease management due to its anatomical complexity and heterogeneous patterns of regional spread [1,2]. The management of the clinically node-negative (cN0) neck in maxillary SCC remains controversial. Historically, the perceived risk of cervical metastasis in maxillary tumors was considered lower than in other oral cavity subsites, leading many clinicians to favor a watchful waiting approach. However, more recent studies have reported non-negligible rates of occult cervical metastases and regional failure, particularly in advanced T-stage disease and in tumors with aggressive pathological features. These findings have prompted increasing interest in elective neck dissection (END) as a potential strategy to improve regional control and survival outcomes [3,4]. While elective treatment of the neck is well-established in several oral cavity cancers, the evidence supporting END in maxillary SCC is less robust and largely derived from retrospective single-institution series and population-based analyses. Moreover, published studies have reported conflicting results regarding the survival benefit of END compared with observation in patients presenting with a clinically node-negative neck. This uncertainty has resulted in significant variability in clinical practice and guideline recommendations [5,6,7]. Previous studies have reported occult cervical metastasis rates ranging from approximately 14% to over 20% in selected MSCC populations, particularly in advanced T-stage tumors. However, available evidence remains fragmented, largely retrospective, and heterogeneous regarding tumor subsites, inclusion criteria, and neck management strategies. Consequently, the true survival impact of elective neck dissection in clinically node-negative MSCC remains insufficiently defined. To date, no clear consensus exists regarding the optimal management of the cN0 neck in maxillary SCC. A comprehensive synthesis of the available evidence focusing specifically on survival outcomes is therefore warranted. The present systematic review and meta-analysis aimed to evaluate the impact of elective neck dissection compared with observation on overall survival in patients with clinically node-negative maxillary squamous cell carcinoma.

## 2. Materials and Methods

### 2.1. Study Protocol

This systematic review and meta-analysis was conducted in accordance with the Preferred Reporting Items for Systematic Reviews and Meta-Analyses (PRISMA) 2020 guidelines [8] (see Supplementary Materials). The study aimed to evaluate the impact of elective neck dissection (END) on survival outcomes in patients with clinically node-negative maxillary squamous cell carcinoma. The protocol of this review was registered in the International Prospective Register of Systematic Reviews (PROSPERO) under registration number CRD420261345038 and predefined the research question, eligibility criteria, search strategy, outcomes of interest, and methods for data synthesis, ensuring methodological transparency and minimizing the risk of reporting bias. Minor protocol amendments were made after registration; these did not affect the eligibility criteria, outcomes of interest, or planned data synthesis and are publicly available in the PROSPERO record. The study followed the Population, Intervention, Comparison, Outcome (PICO) framework: Population: patients with maxillary squamous cell carcinoma and clinically N0 neck; Intervention: elective neck dissection; Comparison: observation/watchful waiting of the neck; Outcome: overall survival (primary outcome). Secondary outcomes included regional recurrence and occult cervical metastasis when available. Two reviewers independently performed study selection, data extraction, and risk-of-bias assessment. Any discrepancies were resolved by discussion, and when necessary, by consultation with a third reviewer.

### 2.2. Search Strategy

A comprehensive literature search was conducted to identify studies evaluating the management of the clinically node-negative (cN0) neck in maxillary squamous cell carcinoma. The search strategy combined controlled vocabulary terms with free-text keywords related to maxillary neoplasms, squamous cell carcinoma, and elective neck management. The following electronic databases were systematically searched from their inception to March 2026: PubMed/MEDLINE, Embase, and Scopus. Searches were limited to human studies, and no restrictions on publication date were applied. Controlled vocabulary terms (MeSH in PubMed and Emtree in Embase) were combined with free-text keywords searched within titles and abstracts to maximize sensitivity. The electronic search was adapted for each database. The strings were as follows: PubMed/MEDLINE: (Maxillary Neoplasms[MeSH] OR Maxillary Sinus Neoplasms[MeSH] OR maxilla* OR maxillary OR upper jaw OR maxillary sinus) AND (Carcinoma, Squamous Cell[MeSH] OR squamous cell carcinoma OR SCC) AND (Neck Dissection[MeSH] OR Elective Neck Dissection OR selective neck dissection OR neck management OR occult metastasis OR clinically N0). Embase: (maxilla tumor/exp OR maxillary sinus tumor/exp OR maxilla* OR maxillary OR upper jaw OR maxillary sinus) AND (squamous cell carcinoma/exp OR squamous cell carcinoma OR scc) AND (neck dissection/exp OR elective neck dissection OR selective neck dissection OR neck management OR occult metastasis OR clinically n0). Scopus: TITLE-ABS-KEY((maxilla* OR maxillary OR upper jaw OR maxillary sinus) AND (squamous cell carcinoma) AND (elective neck dissection OR selective neck dissection OR neck dissection)). The full search strategies are also provided in the Supplementary Materials.

### 2.3. Study Selection and Data Collection Process

All records identified through the database searches were imported into EndNote Web Clarivate Analytics, Philadelphia, PA, USA (https://access.clarivate.com/login?app=endnote, accessed on 5 March 2026) for management and duplicate removal. Both automated and manual deduplication procedures were performed prior to screening. Study selection was conducted in two sequential phases. First, titles and abstracts were independently screened by two reviewers to identify potentially eligible studies according to the predefined inclusion criteria. Articles deemed irrelevant were excluded at this stage. In the second phase, the full texts of the remaining articles were independently assessed for eligibility. Studies that did not meet the inclusion criteria were excluded, and the reasons for exclusion were recorded. Data extraction was performed independently by two reviewers using a standardized data collection form. Extracted variables included: study characteristics (first author, year, country, study design), patient population and sample size, tumor subsite and T stage, neck management strategy (elective neck dissection vs. observation), follow-up duration and overall survival outcomes (hazard ratios, survival rates, or Kaplan–Meier data). Any discrepancies during study selection or data extraction were resolved through discussion and consensus. When necessary, a third reviewer was consulted.

### 2.4. Outcomes and Definitions

The primary outcome of this systematic review and meta-analysis was overall survival (OS). When available, hazard ratios (HRs) with corresponding 95% confidence intervals (CIs) comparing elective neck dissection (END) versus observation were extracted. When HRs were not directly reported, they were estimated from Kaplan–Meier curves or other available survival data when feasible. Secondary outcomes included: occult cervical metastasis rate, defined as the proportion of pathologically positive lymph nodes identified after elective neck treatment in clinically node-negative patients; regional recurrence, defined as the occurrence of cervical nodal disease during follow-up in initially node-negative patients. For studies reporting multiple survival analyses, adjusted HRs from multivariable models were preferentially extracted when available. When both adjusted and unadjusted estimates were reported, the adjusted estimates were used for the primary meta-analysis. Elective neck dissection (END) was defined as any planned surgical neck dissection performed in the absence of clinically or radiologically evident nodal disease. Observation (watchful waiting) was defined as the absence of elective neck treatment with clinical and/or radiologic surveillance of the neck.

### 2.5. Risk of Bias Assessment

The methodological quality of the included studies was assessed using the Risk Of Bias In Non-randomized Studies of Interventions (ROBINS-I) tool [9]. Two reviewers independently evaluated each study across the following bias domains: bias due to confounding, bias in selection of participants, bias in classification of interventions, bias due to deviations from intended interventions, bias due to missing data, bias in measurement of outcomes, and bias in selection of the reported result. Each domain was judged as low, moderate, serious, or critical risk of bias according to ROBINS-I guidance. An overall risk-of-bias judgment was then assigned to each study. Any disagreements between reviewers were resolved through discussion and consensus, with involvement of a third reviewer when necessary.

### 2.6. Statistical Analysis

The primary effect measure was the hazard ratio (HR) for overall survival comparing elective neck dissection (END) with observation. For each study, log(HR) values and corresponding standard errors (SEs) were extracted or calculated. When HRs and 95% confidence intervals (CIs) were directly reported, they were used for the primary analysis. When not explicitly available, HRs were estimated from Kaplan–Meier curves or other reported survival data using established methods when feasible. Statistical analyses were performed using R version 4.4.1 (R Foundation for Statistical Computing, Vienna, Austria) and the metafor package version 4.6-0. Hazard ratios (HRs) with corresponding 95% confidence intervals (CIs) were extracted from the included studies or calculated from reported survival data when necessary. A random-effects meta-analysis model was applied to estimate pooled hazard ratios for overall survival, accounting for potential between-study variability. Heterogeneity was interpreted according to Cochrane recommendations, considering both the magnitude and uncertainty of I^2^ estimates together with clinical and methodological heterogeneity. Potential publication bias was visually evaluated using funnel plots, and sensitivity analysis was conducted using a leave-one-out approach to assess the influence of individual studies on the pooled estimates. Given the limited number of studies available for quantitative synthesis, leave-one-out analysis was considered the most appropriate sensitivity approach.

Publication bias was assessed through the visual inspection of funnel plots, given the limited number of included studies. The certainty of evidence for the primary outcome was assessed using the GRADE (Grading of Recommendations Assessment, Development and Evaluation) framework. Prediction intervals were not calculated because of the limited number of studies and the low observed heterogeneity.

## 3. Results

### 3.1. Study Selection

The literature search identified 791 records from electronic databases (PubMed n = 388, Scopus n = 146, Embase n = 257). After the removal of duplicates (n = 321), 470 records remained for title and abstract screening. Following the screening process, 44 articles were assessed for full-text eligibility. Of these, 30 articles were excluded for the following reasons: case reports (n = 3), conference abstracts (n = 1), maxillary cases not isolable (n = 4), lack of comparison between elective neck dissection and observation (n = 21), and non-original studies such as reviews (n = 1). Ultimately, 14 studies met the predefined inclusion criteria and were included in the qualitative synthesis. Among these studies, five provided sufficient time-to-event data for quantitative synthesis and were included in the meta-analysis. Potential overlap among SEER-based populations was carefully evaluated by comparing study periods, inclusion criteria, tumor subsites, and analyzed cohorts. Given the differences in study design and patient selection, all studies were retained for qualitative synthesis; however, only studies providing non-overlapping or methodologically distinct survival analyses were included in the quantitative meta-analysis.

**Figure 1:**
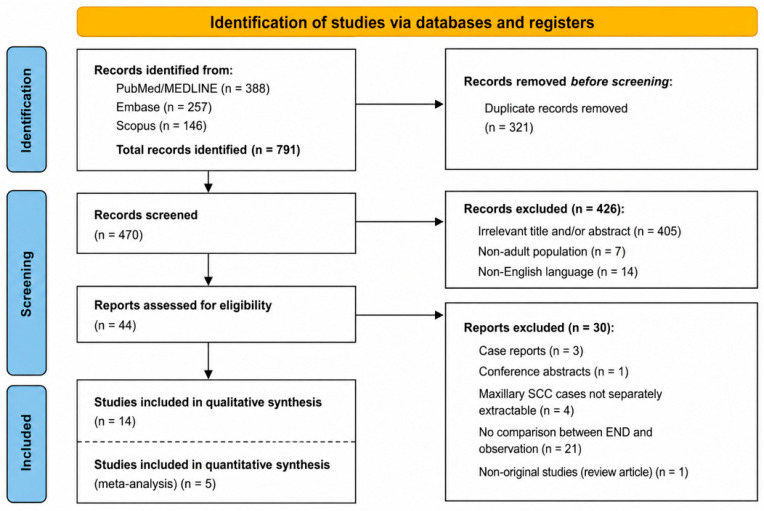
PRISMA flow diagram illustrating the study selection process according to the PRISMA 2020 guidelines, including the identification, screening, eligibility, and inclusion of studies for the qualitative and quantitative synthesis.

The study selection process is illustrated in the PRISMA flow diagram (Figure 1).

### 3.2. Study Characteristics

The main characteristics of the 14 included studies are summarized in Table 1.

The studies were published between 2012 and 2024 and were predominantly retrospective observational cohorts. Overall, they evaluated patients with maxillary squamous cell carcinoma presenting with a clinically node-negative (cN0) neck. Tumor subsites included the maxillary gingiva, alveolus, hard palate, and maxillary sinus. Patients underwent either elective neck dissection (END) or observation with clinical and/or radiologic surveillance of the neck.

Follow-up duration and reporting of oncologic outcomes varied among studies. While several studies reported overall survival using hazard ratios from Cox regression analyses, others reported survival through Kaplan–Meier curves or descriptive survival rates.

### 3.3. Risk of Bias Results

Overall, the included studies showed predominantly moderate to serious risk of bias. The main source of bias was confounding related to the non-randomized allocation of elective neck dissection, often influenced by tumor stage, institutional practice, or surgeon preference. Large registry-based studies using multivariable adjustment or propensity score matching were judged to have moderate overall risk of bias, whereas small retrospective single-center or non-comparative studies were generally judged to have serious risk of bias.

**Figure 2:**
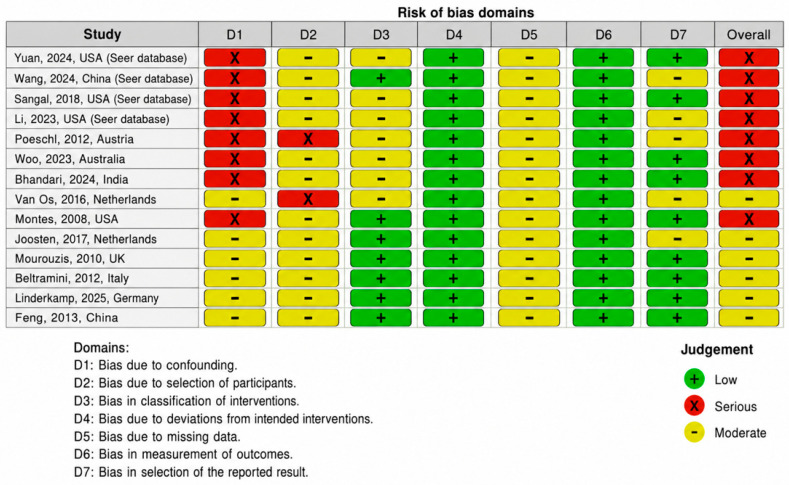
Risk of bias assessment of included studies using the ROBINS-I tool [10,11,12,13,14,15,16,17,18,19,20,21,22,23].

The detailed risk-of-bias assessment is summarized in Figure 2.

### 3.4. Meta-Analysis of Overall Survival

**Figure 3:**
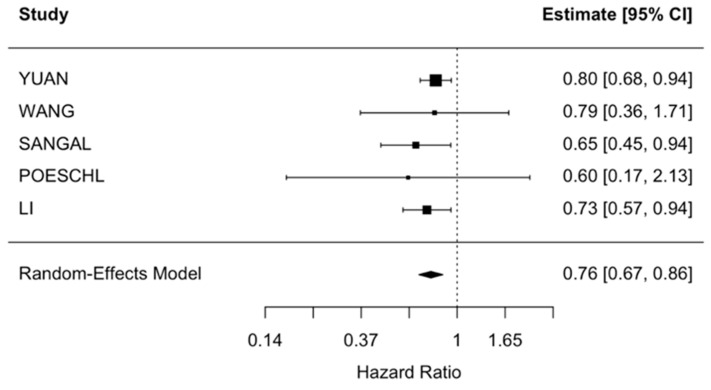
Forest plot of pooled hazard ratios for overall survival comparing elective neck dissection versus observation [10,11,12,13,14].

Five studies reporting hazard ratios for overall survival comparing elective neck dissection versus observation were included in the quantitative synthesis. The pooled analysis using a random-effects model demonstrated that elective neck dissection was associated with significantly improved overall survival compared with observation (HR = 0.76, 95% CI 0.67–0.86; *p* < 0.001). Statistical heterogeneity across studies was negligible, with an I^2^ value of 0% and a non-significant Cochran’s Q test (*p* = 0.85). Individual and pooled hazard ratios are presented in the forest plot (Figure 3). Reported occult cervical metastasis rates ranged from approximately 14% to 21% across included studies. Regional recurrence was consistently associated with poorer oncologic outcomes and lower salvageability. Due to substantial heterogeneity in study design, patient selection, and outcome reporting, pooled quantitative analysis of these secondary outcomes was not feasible.

### 3.5. Sensitivity Analysis

Leave-one-out sensitivity analysis demonstrated that the pooled estimate remained stable after sequential removal of each individual study, confirming the robustness of the meta-analysis results.

### 3.6. Publication Bias

**Figure 4:**
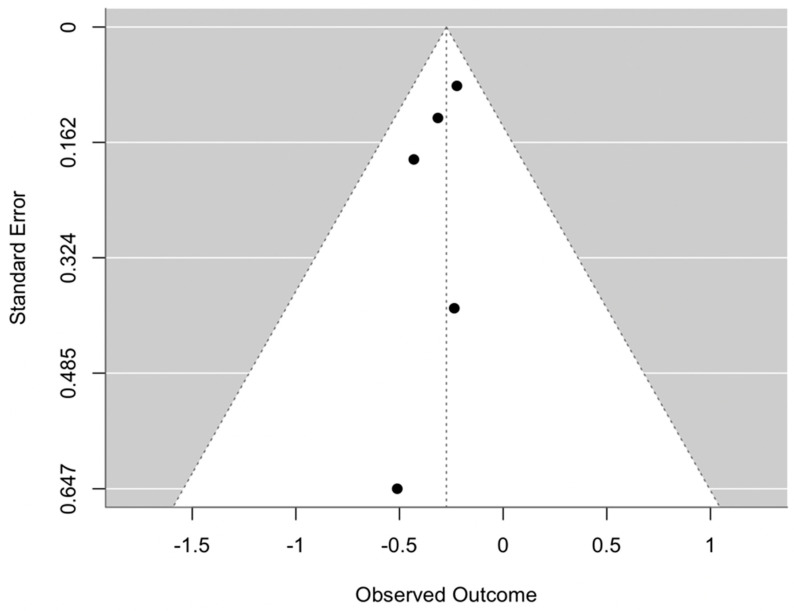
Funnel plot assessing potential publication bias.

Visual inspection of the funnel plot did not suggest marked asymmetry. However, interpretation is limited due to the small number of included studies (<10) (Figure 4).

## 4. Discussion

The optimal management of the clinically node-negative (cN0) neck in patients with maxillary squamous cell carcinoma remains a matter of debate. Unlike other oral cavity subsites, maxillary tumors have historically been considered to carry a lower risk of cervical metastasis due to the distinct regional metastatic patterns of the maxilla. However, more recent evidence suggests that the incidence of occult nodal metastasis may be higher than previously assumed, raising questions about the role of elective neck dissection (END) in this patient population. In the present systematic review, a total of 14 studies evaluating the management of the cN0 neck in maxillary squamous cell carcinoma were identified. Among these, five studies provided sufficient survival data for inclusion in the meta-analysis. The pooled analysis demonstrated a survival advantage associated with elective neck dissection, with a pooled hazard ratio for overall survival of 0.76 (95% CI 0.67–0.86). Importantly, heterogeneity across studies was negligible (I^2^ = 0%), indicating a high degree of consistency among the included studies. These findings suggest that elective treatment of the clinically negative neck may reduce the risk of mortality in patients with maxillary squamous cell carcinoma. Several studies included in this review support the oncological benefit of elective neck management. Large population-based analyses have reported that neck dissection represents an independent prognostic factor associated with improved survival outcomes in patients with maxillary oral squamous cell carcinoma [10]. Similarly, retrospective analyses using national cancer registries have shown that elective neck dissection can significantly reduce the hazard of death in patients with maxillary sinus squamous cell carcinoma [11]. Additional SEER-based studies have also demonstrated improved overall survival and cancer-specific survival in patients undergoing END compared with those managed with observation alone [13]. Collectively, these findings suggest that occult nodal metastases may occur in a clinically significant proportion of patients despite the absence of clinically detectable cervical disease. Nevertheless, the survival benefit of END appears to depend on specific tumor characteristics. Several studies have reported that the advantage of elective neck treatment is more pronounced in patients with advanced primary tumors, particularly those with T3 or T4 disease [10,11]. In contrast, the role of END in early-stage tumors remains less clear. For example, some authors have reported that patients with T1–T2 tumors may not derive a significant survival advantage from elective neck dissection and could potentially be managed with careful surveillance strategies [12]. These observations suggest that tumor stage plays a critical role in determining the risk of occult cervical metastasis and therefore the potential benefit of prophylactic neck treatment. In addition to tumor stage, several clinicopathological factors have been associated with an increased risk of occult nodal metastasis in maxillary squamous cell carcinoma. Factors such as depth of invasion, tumor differentiation, and primary tumor location have been proposed as potential predictors of cervical metastasis [15]. In particular, tumors with greater depth of invasion or higher histological grade appear to have a higher propensity for regional spread [24,25]. Identification of these high-risk features may therefore assist clinicians in selecting patients who may benefit most from elective neck treatment while avoiding overtreatment in low-risk cases. Despite these findings, the available evidence remains limited by several methodological considerations. Most of the studies included in this systematic review were retrospective observational studies, which are inherently subject to selection bias and confounding factors. In many cases, the decision to perform elective neck dissection was influenced by surgeon preference, tumor characteristics, or institutional treatment protocols. Furthermore, several studies were based on large administrative databases such as the SEER registry, which, although valuable for population-level analyses, often lack detailed clinicopathological information including depth of invasion, margin status, and patterns of recurrence. Another limitation of the current literature is the relative rarity of maxillary squamous cell carcinoma, which makes prospective studies difficult to conduct. As a result, most available studies include relatively small patient cohorts or heterogeneous populations including tumors arising from different maxillary subsites. Additionally, only a subset of the available studies reported hazard ratios suitable for quantitative synthesis, limiting the number of studies that could be included in the meta-analysis. Nevertheless, the present study provides a comprehensive synthesis of the currently available evidence regarding the management of the cN0 neck in maxillary squamous cell carcinoma. The results of the meta-analysis suggest that elective neck dissection could be associated with improved survival, particularly in patients with higher-risk disease characteristics such as advanced tumor stage. These findings support the growing body of literature suggesting that the risk of occult cervical metastasis in maxillary squamous cell carcinoma may be clinically relevant and should be considered during treatment planning. Future research should focus on prospective multicenter studies with standardized reporting of clinicopathological variables, treatment strategies, and survival outcomes. In particular, better stratification according to tumor subsite, T stage, and pathological risk factors may help refine indications for elective neck dissection in clinically node-negative maxillary squamous cell carcinoma.

## 5. Limitations

Several limitations of this study should be acknowledged. First, all included studies were retrospective observational analyses and were therefore inherently susceptible to selection bias, confounding, and treatment allocation bias. In many cases, the decision to perform elective neck dissection was influenced by tumor characteristics, surgeon preference, and institutional treatment protocols. Second, several studies were derived from large population-based databases such as SEER, which, although valuable for evaluating large patient cohorts, lack detailed clinicopathological information. Important prognostic variables, including depth of invasion, margin status, perineural invasion, lymphovascular invasion, and details regarding adjuvant treatment, were not consistently available across studies and therefore could not be reliably incorporated into the pooled analysis. Third, only five studies provided sufficient data for quantitative synthesis, thereby limiting the statistical power of the meta-analysis and precluding more extensive analyses, including subgroup analyses, meta-regression, and a robust assessment of publication bias. Furthermore, maxillary squamous cell carcinoma is a relatively rare malignancy, and the available literature frequently includes heterogeneous patient populations with tumors arising from different maxillary subsites. Such heterogeneity may influence both the biological behavior of the disease and the risk of occult cervical metastasis. In addition, follow-up duration was inconsistently reported across studies and may have affected survival outcome assessment. Despite these limitations, the present study provides a comprehensive synthesis of the currently available evidence regarding the management of the clinically node-negative neck in maxillary squamous cell carcinoma. Nevertheless, the overall certainty of evidence was judged to be low according to the GRADE assessment, primarily because of the retrospective design of the included studies, potential selection bias, and heterogeneity in outcome reporting.

## 6. Conclusions

In conclusion, this systematic review and meta-analysis suggests that elective neck dissection may confer a survival benefit in patients with clinically node-negative maxillary squamous cell carcinoma, with the pooled analysis demonstrating a significant reduction in mortality associated with END. The available evidence indicates that the benefit of elective neck treatment may be particularly relevant in patients with advanced primary tumors or other high-risk features, whereas the role of END in early-stage disease remains less clearly defined. Given the limitations of the currently available evidence, further prospective multicenter studies are warranted to better define the indications for elective neck treatment and to identify patients who may benefit most from prophylactic management of the cN0 neck.

## Figures and Tables

**Table 1:** Features of the 14 included studies.

Author/Year/Country	Study Design	N Cases (Sex/Age)	Tumor Site	N END	N OBS	Follow Up
Yuan, 2024, USA (Seer database) [10]	Retrospective population-based study (SEER database)	2512, 53.4% males, 46.6% females, 70–79 years	Oral maxilla (upper gingiva, hard palate, soft palate, upper lip)	312	2200	NR
Wang, 2024, China (Seer database) [11]	Retrospective population-based study (SEER) with propensity score matching	180, 104 males, 76 females, 67 ± 10 years	Maxillary sinus SCC	40	140	Median 25 months
Sangal, 2018, USA (Seer database) [12]	Retrospective population-based database study	927, 66% males, 34% females, 66.41 ± 14.15 years	Maxillary sinus SCC	146	475	NR
Li, 2023, USA (Seer database) [13]	Retrospective population-based cohort study	777, 62.29% males, 35.71% females, 66.42 ± 13.5 years	Maxillary sinus SCC	273	504	NR
Poeschl, 2012, Austria [14]	Retrospective single-center cohort study	74, 49 males, 25 females, 61 years	Oral maxilla (maxillary alveolus, gingiva, hard palate)	36	38	6–130 months
Woo, 2023, Australia [15]	Retrospective single-center cohort study	77 patients, 49.3% males, 50.7% females, 66 years	Oral maxilla (maxillary alveolus/hard palate)	22	30	51 months
Bhandari, 2024, India [16]	Retrospective single-center cohort study	53 patients, 73.6% males, 60 years	Maxillary alveolus + hard palate SCC	44	9	24 months
Van Os, 2016, Netherlands [17]	Retrospective multicenter cohort study	114 patients, 47 men, 67 women, 64 years	Maxillary alveolus + hard palate SCC	NR	NR	3.2 years
Montes, 2008, USA [18]	Retrospective single-center case series + literature review	14 patients, 5 men, 9 women, 53–90 years	Maxillary Alveolus/Maxillary Gingiva	6	8	16.58 ± 4.23 months
Joosten, 2017, Netherlands [19]	Retrospective single-center cohort study	95 patients, 48% males, 52% females, 70 years	Oral maxilla	18	77	5 years
Mourouzis, 2010, UK [20]	Retrospective single center case series	17 patients, 11 men, 6 women, 43–88 years	Maxillary gingiva, alveolus, hard palate SCC	1	11	NR
Beltramini, 2012, Italy [21]	Retrospective multicenter cohort study	65 patients, 47.6% men, 52.3% women, 68.5 years	Maxillary alveolus/gingiva/hard palate SCC	16	42	43.3 months
Linderkamp, 2025, Germany [22]	Retrospective single center study	17 cN0 patients included in comparative analysis	Alveolar process and hard palate	13	4	NR
Feng, 2013, China [23]	Retrospective single center study	129 patients, 49.6% males, 50.4% women, 63.8 years (among the groups)	Hard palate, alveolar process and maxillary gingiva, soft palate.	50	79	62 months

## Data Availability

The data that support the findings of this study are available from the corresponding author upon reasonable request.

## Supplementary Materials

The following supporting information can be downloaded at: https://www.mdpi.com/article/10.3390/jcm15114310/s1. Supplementary File S1: PRISMA 2020 Checklist; Supplementary File S2: Full search strategies.

## Abbreviations

The following abbreviations are used in this manuscript:

cN0	Clinically Node-Negative Neck
MSCC	Maxillary Squamous Cell Carcinoma
END	Elective Neck Dissection
