# Supplementary material for: Management of the Clinically N0 Neck in Maxillary Squamous Cell Carcinoma: A Systematic Review and Meta-Analysis
Source: J Clin Med. 2026 Jun 2;15(11):4310. doi: 10.3390/jcm15114310 (PMC13257650; doi:10.3390/jcm15114310)
Supplement: Supplementary file 1 [file jcm-15-04310-s001.zip › Supplementary File S2. Full search strategies.pdf]

A comprehensive literature search was conducted in PubMed/MEDLINE, Embase, and Scopus from inception to March 2026.

PubMed/MEDLINE:

(Maxillary Neoplasms[MeSH] OR Maxillary Sinus Neoplasms[MeSH] OR maxilla* OR maxillary OR upper jaw OR maxillary sinus) AND (Carcinoma, Squamous Cell[MeSH] OR squamous cell carcinoma OR SCC) AND (Neck Dissection[MeSH] OR Elective Neck Dissection OR selective neck dissection OR neck management OR occult metastasis OR clinically N0)

Embase:

(maxilla tumor/exp OR maxillary sinus tumor/exp OR maxilla* OR maxillary OR upper jaw OR maxillary sinus) AND (squamous cell carcinoma/exp OR squamous cell carcinoma OR scc) AND (neck dissection/exp OR elective neck dissection OR selective neck dissection OR neck management OR occult metastasis OR clinically n0)

Scopus:

TITLE-ABS-KEY((maxilla* OR maxillary OR upper jaw OR maxillary sinus) AND (squamous cell carcinoma) AND (elective neck dissection OR selective neck dissection OR neck dissection))
